# Rehabilitation plus OnabotulinumtoxinA Improves Motor Function over OnabotulinumtoxinA Alone in Post-Stroke Upper Limb Spasticity: A Single-Blind, Randomized Trial

**DOI:** 10.3390/toxins9070216

**Published:** 2017-07-11

**Authors:** Deidre Devier, JoAnn Harnar, Leandro Lopez, Allison Brashear, Glenn Graham

**Affiliations:** 1 Department of Neurology, 1542 Tulane Ave, Room 111B, New Orleans, LA 70112, USA; 2 Department of Neurology, MSC10 5620, Health Sciences Center. 1 University of New Mexico, Albuquerque, NM 87131-0001, USA; jharnar@salud.unm.edu; 3 New Mexico VA Healthcare System, 1501 San Pedro SE, Albuquerque, New Mexico, NM 87108, USA; leandro.lopez@va.gov; 4 Department of Neurology, Wake Forest University Baptist Medical Center, Medical Center Blvd, Winston Salem, NC 27157, USA; abrashea@wakehealth.edu; 5 VA Medical Center, 4150 Clement St, San Francisco, CA 94121, USA; Glenn.Graham@va.gov

**Keywords:** stroke, rehabilitation, onabotulinumtoxinA, occupational therapy, muscle spasticity, physical therapy

## Abstract

Background: OnabotulinumtoxinA (BoNT-A) can temporarily decrease spasticity following stroke, but whether there is an associated improvement in upper limb function is less clear. This study measured the benefit of adding weekly rehabilitation to a background of BoNT-A treatments for chronic upper limb spasticity following stroke. Methods: This was a multi-center clinical trial. Thirty-one patients with post-stroke upper limb spasticity were treated with BoNT-A. They were then randomly assigned to 24 weeks of weekly upper limb rehabilitation or no rehabilitation. They were injected up to two times, and followed for 24 weeks. The primary outcome was change in the Fugl–Meyer upper extremity score, which measures motor function, sensation, range of motion, coordination, and speed. Results: The ‘rehab’ group significantly improved on the Fugl–Meyer upper extremity score (Visit 1 = 60, Visit 5 = 67) while the ‘no rehab’ group did not improve (Visit 1 = 59, Visit 5 = 59; *p* = 0.006). This improvement was largely driven by the upper extremity “movement” subscale, which showed that the ‘rehab’ group was improving (Visit 1 = 33, Visit 5 = 37) while the ‘no rehab’ group remained virtually unchanged (Visit 1 = 34, Visit 5 = 33; *p* = 0.034). Conclusions: Following injection of BoNT-A, adding a program of rehabilitation improved motor recovery compared to an injected group with no rehabilitation.

## 1. Introduction

While several blinded and open-label studies have demonstrated the ability of botulinum toxin to temporarily decrease spasticity following stroke, as measured by standard assessments such as the Modified Ashworth Scale [1,2,3,4,5,6,7,8], the ability of botulinum toxin to improve upper limb function following stroke is less clear, with some studies [1,3,4,5,6,7,8], though not all [2,7], reporting functional improvement. Two recent meta-analyses of randomized controlled trials demonstrated that botulinum toxin treatment resulted in a moderate improvement in upper limb function [9,10]. Despite large clinical trials [2,3,11] and FDA approval, the exact timing, use of adjunct rehabilitation, and continuation of lifelong botulinum toxin treatment remains unclear [12,13].

A recent Cochrane Review included three randomized clinical trials for post-stroke spasticity involving 91 participants [14]. It aimed to determine the efficacy of multidisciplinary rehabilitation programs following treatment with botulinum toxin, and found some evidence supporting modified constraint-induced movement therapy and dynamic elbow splinting. There have been varied study designs exploring rehabilitation in persons after the injection of botulinum toxin or a placebo [13,15], rehabilitation in persons after the injection of botulinum toxin or no injection [16], or rehabilitation after the injection of botulinum toxin with no control condition [17]. As the use of botulinum toxin expands and is beneficial in reducing spasticity and costs [18], the benefit of adding upper limb rehabilitation continues to be questioned. We designed this multi-center, randomized, single-blind clinical trial to assess improvement in patient sensory and motor outcome following the injection of onabotulinumtoxinA (BoNT-A), comparing the effects of rehabilitation versus no rehabilitation, using the upper extremity portion of the Fugl–Meyer Assessment of Sensorimotor Recovery After Stroke [19] as the primary outcome measure. While patients could not be blinded to their randomization to receive additional rehabilitation versus no rehabilitation, the assessments of all of the outcome measures were performed by evaluators blinded to rehabilitation assignment in this single-blind design.

## 2. Results

**Figure 1:**
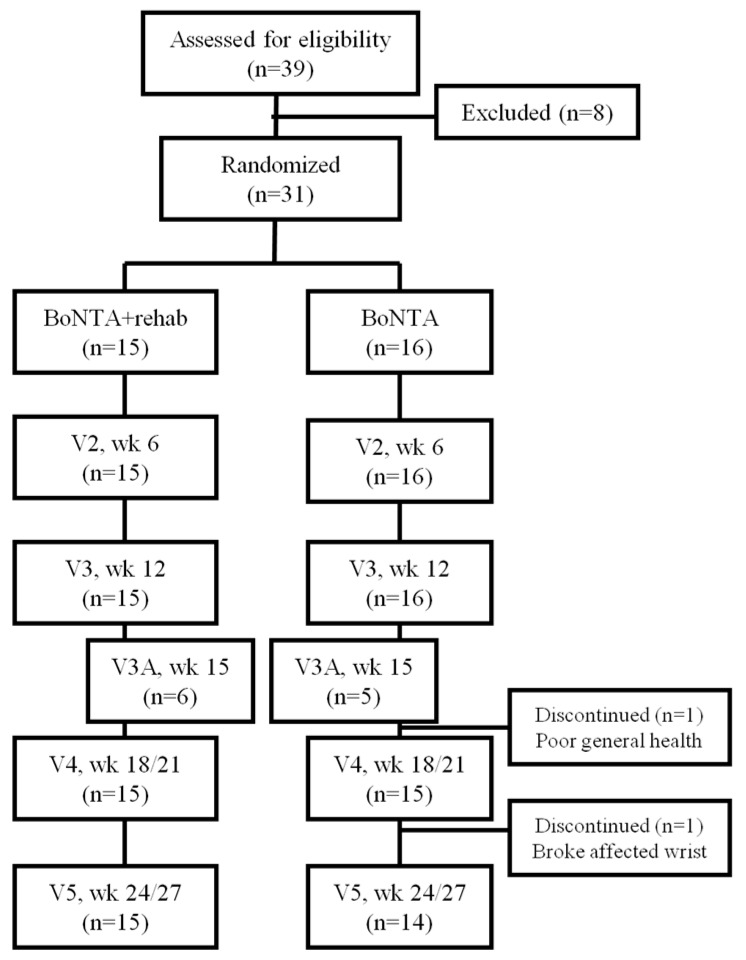
CONSORT Flow Diagram of the Study. BoNTA = onabotulinumtoxinA; V = visit; wk = week.

Thirty-one patients with post-stroke upper limb spasticity were enrolled, with 29 completing the study (Figure 1). The strokes occurred an average of 6 years prior to study entry, with a range of 6 months to 16½ years. The upper extremity postures treated included flexed elbow, pronated forearm, flexed wrist, flexed fingers, and clenched fist, and were evenly distributed between the treatment groups (the initial dose of BoNT-A administered was left up to the clinician’s judgment based on the amount of spasticity present, and did not differ between groups). One participant (‘no rehab’, injected at Visits 1 and 3A) left the study after Visit 3A due to a deterioration in general health and an inability to travel to study visits. A second participant (‘no rehab’, injected at Visits 1 and 3A) left the study after Visit 4 due to a fall with a broken affected wrist. All of the participants were injected at Visit 1, 19 were injected at Visit 3 (8 ‘rehab’; 11 ‘no rehab’), and 7 were injected at Visit 3A (3 ‘rehab’; 4 ‘no rehab’). Those participants who did not receive injections at Visits 3 or 3A had a level of spasticity that either did not meet the injection criteria due to an Ashworth score of <2 in the wrist (and/or fingers) or one that was felt to be too low to warrant injection. Table 1 provides a description of each group with regard to age, sex, race, whether the stroke occurred in the dominant hemisphere, and clinical measures. At baseline, the treatment groups did not differ on any demographic or clinical variables.

### 2.1. Primary Outcome Measure

**Figure 2:**
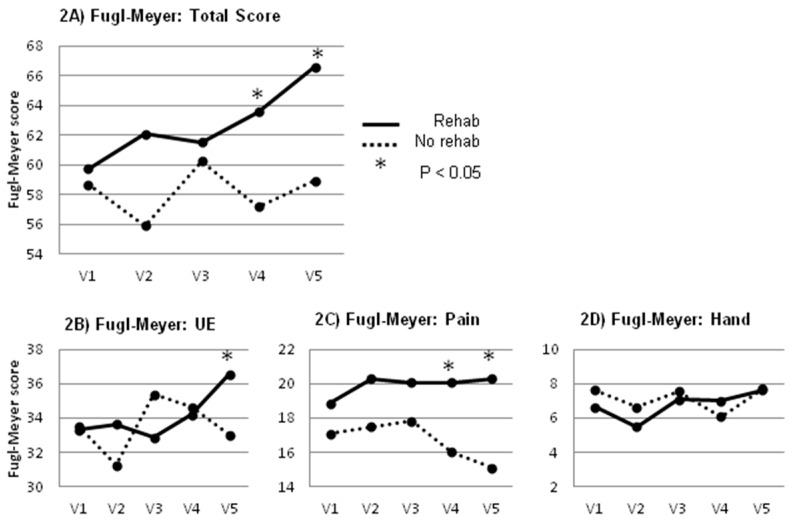
(**A**) Total Fugl–Meyer (F–M) scores across visits 1–5. There was a significant change in F–M score that was driven by the F–M by Group interaction; (**B**) F–M subscale rating passive and active range of motion. There was a significant F–M by Group interaction; (**C**) F–M subscale rating pain during passive range of motion showing a significant F–M by Group interaction; (**D**) F–M subscale rating hand function and grasp. There was a significant overall change in this score in F–M, but no significant interaction.

The Primary Outcome Measure, change in the Fugl–Meyer upper extremity score across all study visits, showed that the ‘rehab’ group significantly improved (Visit 1 = 60, Visit 5 = 67) versus the ‘no rehab’ group that did not improve (Visit 1 = 59, Visit 5 = 59; *p* = 0.006. See Table 2). In post hoc comparisons, the two treatment groups diverged significantly beginning at Visit 3 versus later visits (Figure 2). The same analyses were completed with data using the ‘last observation carried forward’ for missing data (four participants missed one of the five follow-up visits), and the group results were unchanged (data not shown).

The functional and anatomic subscales of the Fugl–Meyer were also analyzed. The upper extremity “movement” subscale showed that the ‘rehab’ group improved (Visit 1 = 33, Visit 5 = 37), while the ‘no rehab’ group remained virtually unchanged (Visit 1 = 34, Visit 5 = 33). This group difference was significant (*p* = 0.034).

The “pain” subscale showed a difference between the groups, with the ‘rehab’ group’s scores remaining fairly stable (Visit 1 = 19, Visit 5 = 20) while the ‘no rehab’ group’s scores worsened slightly (Visit 1 = 17, Visit 5 = 15), also a significant difference (*p* = 0.043).

The “hand, wrist function, and coordination” subscales did not identify any differences between the groups (data not shown).

### 2.2. Secondary Outcome Measures

The Secondary Outcome Measures included the Disability Assessment Scale, which showed no significant differences between the two groups. Both groups self-reported improvement, but the groups did not differ.

**Figure 3:**
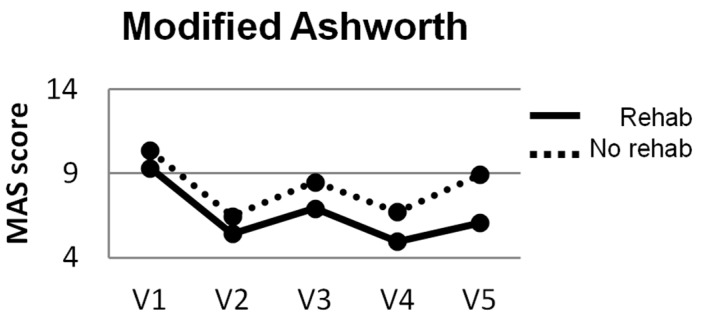
Modified Ashworth scores across visits 1–5. There was a significant change in Ashworth scores, but no Ashworth by Group interaction.

The Ashworth did not measure a difference between the two groups. Both groups had a reduction in spasticity following injection, but the groups did not differ (Table 3). The pattern of changes showed a decrease in muscle tone following injection, but a gradual return of spasticity as the time from injection increased (Figure 3). An Ashworth score of ≥2 makes residual motor activity unlikely and difficult to assess. At the final study assessment, only 3 of 16 participants in the ‘no rehab’ group had spasticity that was ≤2, while 8 of 15 in the ‘rehab’ group fell below that threshold. This finding was likely related to the improved movement measured by the F–M in the rehab group.

Fischer’s Exact Test found no significant differences between treatment groups in those who were not re-injected at Visits 3 or 3A due to low levels of spasticity. Although not statistically significant, four of five participants who did not meet re-injection criteria due to low measures of spasticity any time after the first injection were in the ‘rehab’ group. (data not shown).

**A Safety:** Injection of BoNT-A was shown to be safe. There were six serious adverse events reported during the study, but none were reported as being related to the study’s intervention.

## 3. Discussion

This study showed that the injection of BoNT-A reduced spasticity as previously reported [2,3,4,5,6,7,8,20,21,22,23], while the addition of rehabilitation therapy following injection resulted in improved upper limb motor function and activity. Previous outcomes studied have often used objective measurements of spasticity and subjective self-reports of disability or the achievement of participant-identified goals. Similar to these previous reports, the current study measured a change in spasticity and self-reports, but these changes were unrelated to rehabilitation.

The majority of previous studies of botulinum toxins have not included adjunctive rehabilitation. Rather, they have prohibited changes in the participants’ treatment regimens throughout the study period. While some studies have objectively measured functional outcomes [1,2,4,5,7,21], the process is often time consuming, the assessment scales and the items measured vary greatly from one study to another, and some items are insensitive to change in upper limb function. Wolf et al. designed a study to determine the extent to which botulinum toxin combined with a standardized exercise program could improve upper extremity function and quality of life [15]. Their participants were randomized to receive either an injection of botulinum toxin or saline followed by 12–16 exercise sessions. They did not find botulinum toxin plus rehabilitation to be superior to placebo plus rehabilitation in their primary outcome measure. However, the combination of botulinum toxin with a targeted rehabilitation program versus botulinum toxin without rehabilitation is untested in the stroke literature.

The data from this study suggest that adjunctive rehabilitation leads to improved outcomes in motor function/activity as measured by the upper extremity portion of the Fugl–Meyer. This Fugl–Meyer assessment differs from self-reports in that it was administered by blinded therapists and offers a more objective measurement of motor function/activity. The temporal trend in the data further suggests that the continuation of rehabilitation following the injection of BoNT-A may lead to even greater gains in motor function over time. The two treatment groups appeared to be on different trajectories at the final Fugl–Meyer assessment, with the ‘rehab’ group experiencing an upward trend in upper extremity recovery of motor function/activity.

Somewhat surprisingly, the changes in the Fugl–Meyer motor assessment occurred despite a lack of group differences in spasticity scores as measured by the Ashworth scale score. This demonstrates that the additional benefit of occupational or physical therapy was distinct from any further reduction in muscle tone. This finding underscores the importance of using scales sensitive to functional performance to examine the benefits of treatment.

In the current study, the rehabilitation was tailored to the needs and abilities of each research participant, and was administered in an individual setting with instructions for continued work at home between visits. Rehabilitation interventions included techniques such as the electrical stimulation of the affected limb, and passive, assisted, and active range of motion. Additionally, to improve functional capacity, the movements included repetitive exercises such as functional reaching using proprioceptive neuromuscular facilitation patterns. The combination of BoNT-A plus these rehabilitation interventions resulted in improved upper extremity function/activity.

Limitations One limitation of this study is the single-blind design. Admittedly, a double-blind design is best, but the participants could not be blind to the assignment of rehabilitation. However, the primary functional outcome measure (Fugl–Meyer) and the measure of spasticity (Ashworth) were both conducted by blinded raters, who were queried, and there were no instances of unblinding. A placebo controlled trial was not considered, as research has clearly demonstrated that botulinum toxin reduces muscle tone, whereas the question in this study related to the effect of rehabilitation. Further, as all patients had significant chronic motor impairment (6 or more months post-stroke), it would be difficult to include a placebo, “natural-history” arm, since many patients would be reluctant to participate if they could be randomized to no change in care. The inclusion of a third cohort would also increase the required sample size. Finally, the study involved a relatively small sample size, limited functional assessment, and a relatively short follow-up duration. The results need to be confirmed in a larger trial with multiple injections over a longer period of time to determine if the benefits of rehabilitation following injection of BoNT-A continue.

## 4. Conclusions

Rehabilitation following the injection of onabotulinumtoxinA for post-stroke upper limb spasticity resulted in improved functional motor recovery compared to no rehabilitation. This improved outcome was measured over approximately seven months and may continue beyond that time.

## 5. Materials and Methods

### 5.1. Recruitment and Selection

Adult patients with post-stroke upper limb spasticity were recruited from neurology clinics and through local advertisements. The inclusion criteria were: stroke at least 6 months prior to enrollment, focal spasticity in an upper limb with a Modified Ashworth Scale (Ashworth) of 3 or greater in the wrist and/or fingers [24], functional impairment on the Disability Assessment Scale secondary to spasticity, and a minimum weight of 44 kg in order to tolerate the minimum required BoNT-A dosage of 200 U. The exclusion criteria were: an uncontrolled clinically significant medical condition, a known allergy to the study medication, pregnancy, breast-feeding, or planned pregnancy during the study, a fixed contracture or profound atrophy in the spastic limb, prior or current treatment with surgery or neurolytic agents, concurrent rehabilitation that could not be altered to the study treatment plan, a medical condition that put the participant at increased risk with exposure to botulinum toxin, or concurrent treatment with agents affecting neuromuscular transmission.

This study protocol was approved by the two trial sites’ local institutional review boards, and all participants provided signed, written, informed consent and authorization for use and release of health and research study information. The study was conducted in accordance with institutional guidelines. The study was approved by the University of New Mexico Human Research Review Committee, the New Mexico VA Healthcare System Research and Development Committee, and the Wake Forest University Health Sciences Institutional Review Board IRB 00008073.

### 5.2. Baseline

Participants were evaluated at baseline for spasticity and functional abilities. They were then injected with BoNT-A into four required finger and wrist muscles, and additional optional targets using previously described methods [3,6]. All of the injections were performed under electromyography (EMG) guidance of muscle selection and used a BoNT-A concentration of 50 U per mL (standard 2:1 dilution ratio in 0.9% normal saline).

### 5.3. Random Allocation

Each site had a randomization schedule for study subject allocation to one of two groups (rehab or no rehab). Participants were assigned to their treatment group in order of enrollment based on the next allocation on the randomization list.

### 5.4. Additional Visits

Following injection, participants were seen every 6 weeks thereafter. If, by Visit 3 (week 12), upper limb spasticity had reached an Ashworth score of ≥2 in the wrist and/or fingers, participants were eligible for a second injection. Those who did not meet the re-injection criteria by Visit 3 were evaluated for injection 3 weeks later at Visit 3A (week 15). If they did not meet the re-injection criteria at Visit 3A, they did not receive another injection, but were followed through to Visit 5 (final visit, week 27).

Participants randomized to the ‘rehab’ group were scheduled for 24 weekly rehabilitation appointments to begin within 2 weeks of the first injection. Rehabilitation performed by a physical therapist and/or an occupational therapist was tailored to each participant and was designed to be conducted individually or in a small group setting. While at the rehabilitation center, interventions were conducted for 1.5 h and included techniques such as electrical stimulation of the affected limb, and either a passive, active assisted, or full active range of motion. Rehabilitation also included repetitive exercises such as functional reaching using proprioceptive neuromuscular facilitation patterns. In addition, participants were instructed to complete a 1 h daily home exercise program between visits. Compliance with the home exercise program was discussed at study visits, but was not systematically monitored. Participants in the ‘no rehab’ group followed the same injection and assessment schedule, but did not participate in rehabilitation and were not assigned a home exercise program. At the time of consent, all of the participants agreed that if they were assigned to the ‘no rehab’ group, they would not start any new rehabilitation or exercise program for arm spasticity until finished with the study.

### 5.5. Assessment Tools

The primary outcome measure was change in the Fugl–Meyer Assessment upper extremity score across all study visits (visits 1–5) in each treatment arm. The Fugl–Meyer has good validity and reliability [19,25,26], and is responsive to recovery over time [27]. The upper extremity subscale includes volitional movement within synergies, mixing synergies, or with little or no synergy, and reflex activity. The Fugl–Meyer was administered by a therapist blind to treatment assignment.

The Disability Assessment Scale is a self-report assessing four domains: hygiene, dressing, limb posture, and pain. Scores for each item range from 0 (no disability) to 3 (severe disability). The scale, assessed at each visit, measures the level of disability following stroke [3,6] with good intra- and inter-rater reliability [11].

The Modified Ashworth Scale, administered at each visit by the blinded injecting physician (administering physicians: GDG or AB), assessed the tone of the wrist and finger flexors at all visits [24], measuring resistance to passive movement from 0 (no increase in tone) to 4 (limb rigid in flexion or extension), and has good intra and inter-rated reliability [11].

Safety: Adverse events were assessed by the study investigators at each follow-up visit. A serious adverse event was defined as an event that was fatal, life-threatening, permanently disabling, or that required prolonged hospitalization. Vital signs were assessed at every visit.

Blinding: The therapists assessing the primary outcome measure (Fugl–Meyer) and the physicians assessing spasticity (Ashworth) were blind to treatment assignment (‘rehab’ versus ‘no rehab’).

### 5.6. Statistical Analysis

Demographic and clinical variables were analyzed using Chi Square, Fisher’s Exact, and analysis of variance (ANOVA) to test for differences between the treatment groups at baseline using SPSS (IBM SPSS Statistics for Windows, Version 22.0. IBM Corp: Armonk, NY, USA). In addition, after normal distributions were assessed using the Kolmogorov–Smirnov test, repeated measures ANOVA was used with ‘time’ (visits 1–5) as the within-subjects factor and ‘group’ (‘rehab’ versus ‘no rehab’) as the between-subjects factor to compare the effects of the Time by Group interactions. Results were conditioned on significant *F*-values (*p* <0.05), and post hoc analyses were conducted and corrected for multiple comparisons with Tukey HSD tests [28].

## Figures and Tables

**Table 1:** Overview of study design and time line. Any participants who did not meet the re-injection criteria by Visit 3 (week 12) were re-evaluated in another 3 weeks, Visit 3A. Participants randomized to BoNT-A plus rehabilitation had weekly rehabilitation visits.

Visit	Screen/Physical	Fugl–Meyer	Ashworth	Self-Reports	FIM	Inject
Screen	X		X	X		
V1, Wk0		X	X	X	X	X
V2, Wk6		X	X	X		
V3/3A, Wk12/15		X	X	X		X
V4, Wk18/20		X	X	X		
V5, Wk24/27		X	X	X	X	

**Table 2:** Patient characteristics at baseline, comparing the BoNT-A+Rehab with the BoNT-A no rehab group. The groups did not differ on any baseline demographic or clinical variables. The Ashworth scale is the total across elbow, wrist, fingers, and thumb with a possible range of 0–16. Functional Independence Measure (FIM); Disability Assessment Scale (DAS); Patient Disability Scale (PDS); Visual Analog Scale (VAS).

Demographic and Clinical Variables	BoNT-A + Rehab (*n* = 15)	95% CI	BoNT-A no Rehab (*n* = 16)	95% CI	*p* Value
Age, year	58.0 ± 6.6	54.40–61.67	60.9 ± 11.0	55.07–66.81	0.384
Sex, M/F	11/4		10/6		0.704
Stroke in dominant Hemisphere * n/%	5/33%		8/50%		0.473
Race (Caucasian, African American, Hispanic)	10/3/2		11/4/1		0.782
Fugl–Meyer	58.5 ± 12.9	51.39–65.67	58.1 ± 15.6	49.75–66.38	0.928
FIM, motor subscale	70.9 ± 15.2	62.43–79.3	73.5 ± 17.5	64.16–82.84	0.659
Ashworth	9.3 ± 2.8	7.79–10.88	10.1 ± 2.7	8.67–11.58	0.431
DAS	6.9 ± 2.9	5.32–8.55	6.5 ± 2.2	5.33–7.67	0.642
PDS	18.2 ± 6.4	14.68–21.72	18.0 ± 5.1	15.26–20.74	0.924
VAS	1.9 ± 2.8	0.29–3.44	1.3 ± 2.1	0.19–2.41	0.530

* Presumed dominant hemisphere based on hand preference.

**Table 3:** Modified Ashworth scales for each group at every time point.

Average Modified Ashworth Scores	Rehab	No Rehab
Baseline	10.6	10.8
V1	9.3	10.1
V2	5.4	5.7
V3	8.6	6.0
V4	5.0	6.3
V5	6.1	8.9

## References

[1] Sampaio C., Ferreira J.J., Pinto A.A., Crespo M., Ferro J.M., Castro-Caldas A. (1997). Botulinum toxin type a for the treatment of arm and hand spasticity in stroke patients. Clin. Rehabil..

[2] Bakheit A.M., Pittock S., Moore A.P., Wurker M., Otto S., Erbguth F., Coxon L. (2001). A randomized, double-blind, placebo-controlled study of the efficacy and safety of botulinum toxin type a in upper limb spasticity in patients with stroke. Eur. J. Neurol..

[3] Brashear A., Gordon M.F., Elovic E., Kassicieh V.D., Marciniak C., Do M., Lee C.H., Jenkins S., Turkel C., Botox Post-Stroke Spasticity Study Group (2002). Intramuscular injection of botulinum toxin for the treatment of wrist and finger spasticity after a stroke. N. Engl. J. Med..

[4] Pandyan A.D., Vuadens P., van Wijck F.M., Stark S., Johnson G.R., Barnes M.P. (2002). Are we underestimating the clinical efficacy of botulinum toxin (type a)? Quantifying changes in spasticity, strength and upper limb function after injections of botox to the elbow flexors in a unilateral stroke population. Clin. Rehabil..

[5] Rousseaux M., Kozlowski O., Froger J. (2002). Efficacy of botulinum toxin a in upper limb function of hemiplegic patients. J. Neurol..

[6] Gordon M.F., Brashear A., Elovic E., Kassicieh D., Marciniak C., Liu J., Turkel C., Group B.P.S.S. (2004). Repeated dosing of botulinum toxin type a for upper limb spasticity following stroke. Neurology.

[7] Slawek J., Bogucki A., Reclawowicz D. (2005). Botulinum toxin type a for upper limb spasticity following stroke: An open-label study with individualised, flexible injection regimens. Neurol. Sci..

[8] Bhakta B.B., Cozens J.A., Bamford J.M., Chamberlain M.A. (1996). Use of botulinum toxin in stroke patients with severe upper limb spasticity. J. Neurol. Neurosurg. Psychiatry.

[9] Foley N., Pereira S., Salter K., Fernandez M.M., Speechley M., Sequeira K., Miller T., Teasell R. (2013). Treatment with botulinum toxin improves upper-extremity function post stroke: A systematic review and meta-analysis. Arch. Phys. Med. Rehabil..

[10] Baker J.A., Pereira G. (2015). The efficacy of botulinum toxin a for limb spasticity on improving activity restriction and quality of life: A systematic review and meta-analysis using the grade approach. Clin. Rehabil..

[11] Brashear A., Zafonte R., Corcoran M., Galvez-Jimenez N., Gracies J.M., Gordon M.F., McAfee A., Ruffing K., Thompson B., Williams M. (2002). Inter- and intrarater reliability of the ashworth scale and the disability assessment scale in patients with upper-limb poststroke spasticity. Arch. Phys. Med. Rehabil..

[12] Teasell R., Foley N., Pereira S., Sequeira K., Miller T. (2012). Evidence to practice: Botulinum toxin in the treatment of spasticity post stroke. Top. Stroke Rehabil..

[13] Demetrios M., Gorelik A., Louie J., Brand C., Baguley I.J., Khan F. (2014). Outcomes of ambulatory rehabilitation programmes following botulinum toxin for spasticity in adults with stroke. J. Rehabil. Med..

[14] Demetrios M., Khan F., Turner-Stokes L., Brand C., McSweeney S. (2013). Multidisciplinary rehabilitation following botulinum toxin and other focal intramuscular treatment for post-stroke spasticity. Cochrane Database Syst. Rev..

[15] Wolf S.L., Milton S.B., Reiss A., Easley K.A., Shenvi N.V., Clark P.C. (2012). Further assessment to determine the additive effect of botulinum toxin type a on an upper extremity exercise program to enhance function among individuals with chronic stroke but extensor capability. Arch. Phys. Med. Rehabil..

[16] Shaw L., Rodgers H., Price C., van Wijck F., Shackley P., Steen N., Barnes M., Ford G., Graham L., Bo T.I. (2010). Botuls: A multicentre randomised controlled trial to evaluate the clinical effectiveness and cost-effectiveness of treating upper limb spasticity due to stroke with botulinum toxin type a. Health Technol. Assess..

[17] Sun S.F., Hsu C.W., Sun H.P., Hwang C.W., Yang C.L., Wang J.L. (2010). Combined botulinum toxin type a with modified constraint-induced movement therapy for chronic stroke patients with upper extremity spasticity: A randomized controlled study. Neurore. Neural Repair.

[18] Abogunrin S., Hortobagyi L., Remak E., Dinet J., Gabriel S., Bakheit A.M. (2015). Budget impact analysis of botulinum toxin a therapy for upper limb spasticity in the united kingdom. Clin. Outcomes Res..

[19] Fugl-Meyer A.R., Jaasko L., Leyman I., Olsson S., Steglind S. (1975). The post-stroke hemiplegic patient. 1. A method for evaluation of physical performance. Scand. J. Rehabil. Med..

[20] De Boer K.S., Arwert H.J., de Groot J.H., Meskers C.G., Mishre A.D., Arendzen J.H. (2008). Shoulder pain and external rotation in spastic hemiplegia do not improve by injection of botulinum toxin a into the subscapular muscle. J. Neurol. Neurosurg. Psychiatry.

[21] Lim J.Y., Koh J.H., Paik N.J. (2008). Intramuscular botulinum toxin-a reduces hemiplegic shoulder pain: A randomized, double-blind, comparative study versus intraarticular triamcinolone acetonide. Stroke.

[22] Mayer N.H., Whyte J., Wannstedt G., Ellis C.A. (2008). Comparative impact of 2 botulinum toxin injection techniques for elbow flexor hypertonia. Arch. Phys. Med. Rehabil..

[23] Francis H.P., Wade D.T., Turner-Stokes L., Kingswell R.S., Dott C.S., Coxon E.A. (2004). Does reducing spasticity translate into functional benefit? An exploratory meta-analysis. J. Neurol. Neurosurg. Psychiatry.

[24] Ashworth B. (1964). Preliminary trial of carisoprodol in multiple sclerosis. Practitioner.

[25] Duncan P.W., Propst M., Nelson S.G. (1983). Reliability of the fugl-meyer assessment of sensorimotor recovery following cerebrovascular accident. Phys. Ther..

[26] Gladstone D.J., Danells C.J., Black S.E. (2002). The fugl-meyer assessment of motor recovery after stroke: A critical review of its measurement properties. Neurore. Neural Repair.

[27] Duncan P.W., Lai S.M., Keighley J. (2000). Defining post-stroke recovery: Implications for design and interpretation of drug trials. Neuropharmacology.

[28] Hsu J.C. (1996). Multiple Comparisons: Theory and Methods.

